# CD39^+^CD71^+^ erythroid cells exhibit enhanced purine metabolism and T cell–suppressive function in triple-negative breast cancer and other immunoregulatory settings

**DOI:** 10.1093/jimmun/vkag301

**Published:** 2026-11-13

**Authors:** Amirhossein Rahmati, Shima Shahbaz, Mark Hnatiuk, Shokrollah Elahi

**Affiliations:** Division of Foundational Sciences, Mike Petryk School of Dentistry, Faculty of Medicine & Dentistry, University of Alberta, Edmonton, Alberta, Canada; Division of Foundational Sciences, Mike Petryk School of Dentistry, Faculty of Medicine & Dentistry, University of Alberta, Edmonton, Alberta, Canada; Division of Hematology, Faculty of Medicine & Dentistry, University of Alberta, Edmonton, Alberta, Canada; Division of Foundational Sciences, Mike Petryk School of Dentistry, Faculty of Medicine & Dentistry, University of Alberta, Edmonton, Alberta, Canada; Li Ka Shing Institute of Virology, University of Alberta, Edmonton, Alberta, Canada; Women and Children’s Health Research Institute, University of Alberta, Edmonton, Alberta, Canada; Cancer Research Institute of Northern Alberta, University of Alberta, Edmonton, Alberta, Canada; Glycomics Institute of Alberta, University of Alberta, Edmonton, Alberta, Canada; Alberta Transplant Institute, Faculty of Medicine and Dentistry, University of Alberta, Edmonton, Alberta, Canada

**Keywords:** adenosine, CD39, CD73, erythroid progenitors, purine metabolism

## Abstract

CD71^+^ erythroid cells (CECs) expand in tumor-bearing hosts and suppress antitumor immunity, but the mechanisms underlying their immunoregulatory activity remain incompletely understood. Here, we identify CD39-mediated purine metabolism as a previously unrecognized suppressive pathway used by CECs. In the 4T1 model of triple-negative breast cancer, CD39 was enriched on CD45^+^ CECs in the spleen, bone marrow, and tumor microenvironment, whereas CD73 was restricted to a smaller subset. Single-cell RNA sequencing mapped *Entpd1* and *Nt5e* expression across CEC subsets and revealed that CD39^+^ CECs exhibit a broad immunomodulatory transcriptional program. Functionally, CEC-associated CD39 hydrolyzed extracellular ATP to AMP. CD39^+^ CECs, but not their CD39^−^ counterparts, suppressed T cell proliferation, and A2A adenosine receptor blockade partially restored these responses. Exogenous AMP also impaired T cell effector function through adenosine receptor signaling, supporting a model in which CEC-derived AMP promotes suppression through trans-cellular adenosine production and potentially direct receptor engagement. TGF-β increased CD39 and CD73 expression on CECs in association with SMAD3 activation, suggesting a potential reinforcing regulatory circuit. CD39^+^ CECs were also detected during early life, pregnancy, and infections, as well as in human bone marrow, indicating relevance beyond cancer. Collectively, these findings identify the adenosinergic axis as an additional component of the multifactorial suppressive program of CECs and reveal a broader mechanism through which erythroid progenitors regulate immunity across physiological and pathological settings.

## Graphical Abstract

**Graphical Abstract:**
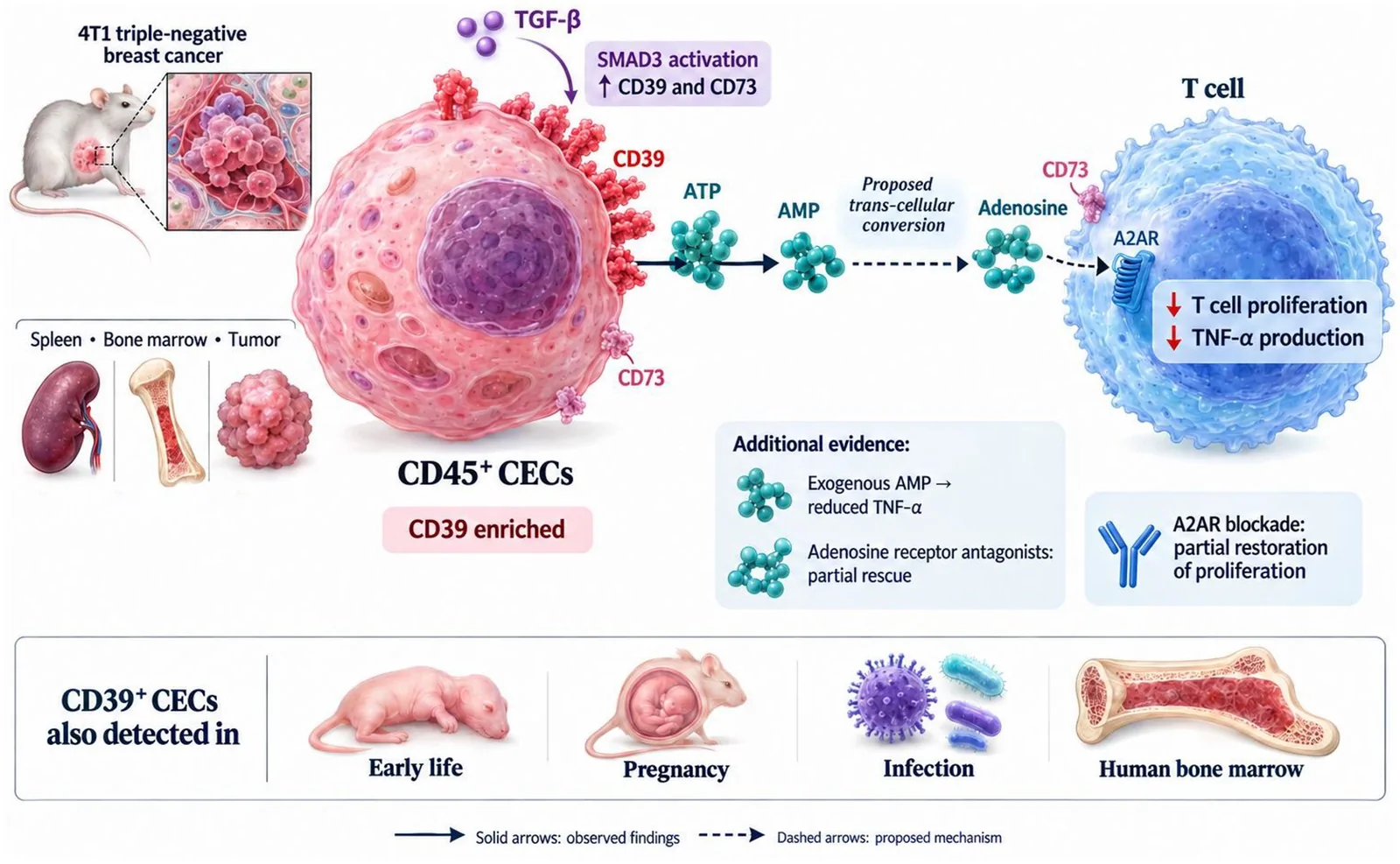

## Introduction

Triple-negative breast cancer (TNBC) accounts for approximately 15% of breast cancers and is pathologically defined by the absence of estrogen receptor, progesterone receptor, and HER2 (human epidermal growth factor receptor 2) expression.^1^^,^^2^ Consequently, TNBC lacks established molecularly targeted therapies, leaving cytotoxic chemotherapy as the primary systemic treatment for this aggressive subtype, which is characterized by earlier onset, higher histological grade, frequent recurrence and a high metastatic potential.^1^ Although combining immune checkpoint inhibitors with chemotherapy has improved outcomes in patients with PD-L1 (programmed cell death-ligand 1)–expressing tumors, durable responses remain limited, and metastatic progression is common.^3–5^ This therapeutic challenge is driven, in large part, by an immunosuppressive tumor microenvironment (TME),^6^ characterized by limited effector T cell infiltration and the accumulation of regulatory T cells (Tregs),^7^^,^^8^ tumor-associated macrophages,^9^ and myeloid-derived suppressor cells (MDSCs).^10^ Identifying the mechanisms that establish and sustain this immunosuppressive environment is therefore critical for developing more effective therapeutic strategies.

CD71^+^ erythroid cells (CECs) comprise erythroid progenitors and precursors identified by the coexpression of CD71 and CD235a in humans and CD71 and TER119 in mice.^11–13^ We previously identified CECs as an abundant immunosuppressive cell population in neonates that suppresses innate and adaptive immune responses, thereby increasing newborn susceptibility to infection.^11^^,^^14^ We and others have since shown that CECs modulate immune responses across diverse physiological and pathological conditions, including pregnancy,^15–17^ bacterial and viral infections,^18–21^ and cancer.^22–25^ CECs mediate immunosuppression through a broad repertoire of mechanisms, including arginase-I/II activity, reactive oxygen species (ROS) production, transforming growth factor-β (TGF-β) signaling, and galectin-9 (Gal-9)/Gal-3 expression, and immune checkpoint molecules such as PD-L1/PD-L2 and VISTA (V-domain Ig Suppressor of T cell Activation).^12^^,^^15^^,^^26–28^ In cancer, tumor-induced extramedullary hematopoiesis drives the expansion and accumulation of CECs in the spleen, where they can outnumber Tregs and MDSCs, and potently suppress CD8+ T cell responses.^22^^,^^23^^,^^25^^,^^29^ CECs therefore represent a functionally diverse immunoregulatory population. However, the complete repertoire of their suppressive mechanisms remains to be defined.

A dominant pathway of tumor immunosuppression is the adenosinergic axis, which is mediated by two ectonucleotidases acting sequentially: CD39 (*Entpd1*) hydrolyzes extracellular ATP and ADP to AMP, and CD73 (*Nt5e*) converts AMP to adenosine, thereby shifting the TME from a proinflammatory to an immunosuppressive state.^30–32^ Adenosine signals mainly through the A2A adenosine receptor (A2AR) on effector T cells, increasing intracellular cAMP and impairing T cell proliferation, cytokine production, and cytotoxicity.^33–35^ Accordingly, genetic deletion or pharmacological blockade of A2AR restores antitumor immunity and limits growth and metastasis of CD73-expressing breast tumors.^36^ Initially characterized in Tregs,^30^ the CD39/CD73 axis is also expressed on MDSCs and macrophages.^31^^,^^37^ However, whether erythroid progenitors contribute to adenosine-mediated immunosuppression remains unknown. Given that CECs are potent immunosuppressive cells that expand in tumor-bearing mice,^12^^,^^22^^,^^24^ and that adenosine signaling is a major driver of immune escape in breast cancer,^36^^,^^38^ we hypothesized that CECs may substantially contribute to their suppressive activity in TNBC through ectonucleotidase-mediated activity.

Here, we investigated the expression and function of CD39 and CD73 on CECs under physiological conditions and in the 4T1 model of TNBC. We found that CD39 is abundantly expressed on CECs under physiological conditions in newborn mice and is further enriched on splenic and bone marrow (BM) CECs in tumor-bearing mice. Notably, CD39 expression was preferentially elevated on the CD45^+^ CEC subset, with expression levels comparable to those observed on Tregs. Additionally, we observed increased expression of CD73 and CD39 on fetal liver and fetal gut CECs, splenic CECs during pregnancy, and in the context of *Bordetella pertussis* infection. Single-cell RNA sequencing (scRNA-seq) delineated the CEC subsets expressing CD39 and CD73, identified alternative adenosine-generating enzymes within these populations, and revealed that CD39^+^ CECs possess a broad immunomodulatory transcriptional program, characterized by elevated TGF-β, arginase, VISTA, and Gal-9. We further demonstrated that CD39 expressed on CECs is enzymatically active, effectively hydrolyzing extracellular ATP to AMP. Functionally, CD39^+^ CECs isolated from tumor-bearing mice suppressed T cell proliferation, an effect that was partially reversed by A2AR blockade. Moreover, AMP, the product of CD39-mediated ATP hydrolysis, was sufficient to impair T cell effector function through adenosine receptor signaling. Collectively, our findings identify CD39 as a functionally important immunosuppressive molecule expressed by CECs.

## Methods

### Ethics statement

All animal experiments were conducted in accordance with the Guide to the Care and Use of Experimental Animals of the Canadian Council on Animal Care. All experimental protocols were approved by the Animal Care and Use Committee at the University of Alberta (Protocol: AUP00001021 and AUP00002737). The study involving human BM aspirates was reviewed and approved by the Health Research Ethics Board of Alberta, Cancer Committee (HREBA#CC-17-0307). Written informed consent was obtained from all participants prior to sample collection.

### Cell line and cell culture

The murine TNBC cell line 4T1 was obtained from the ATCC. Cells were cultured in RPMI 1640 medium (Sigma-Aldrich) supplemented with 10% fetal bovine serum (Sigma-Aldrich) and 1% penicillin/streptomycin (Sigma-Aldrich) and maintained at 37 °C in a humidified incubator with 5% CO_2_.

### Mouse tumor model

Male and female BALB/c mice were purchased from Charles River Laboratories and bred and maintained under pathogen-free conditions in the animal care facility at the University of Alberta. To establish tumors, 5 × 10^5^ 4T1 cells were suspended in 100 µL phosphate-buffered saline (Sigma-Aldrich; #D8537) and injected subcutaneously into the mammary fat pad of female BALB/c mice (8–12 wk old) under isoflurane anesthesia (Sigma-Aldrich; #792632). Tumors became palpable approximately 7 d postinjection, and mice were euthanized for tissue collection 21 d after tumor inoculation.

### Respiratory challenge

*B. pertussis* strain Tohama I was used for infection, as previously described.^18^^,^^39^^,^^40^ Six-day-old neonatal mice were infected intranasally with 25 µL containing 5 × 10^6^ colony-forming units by carefully placing the inoculum at the nares and allowing it to be inhaled. Infected animals were euthanized 4 d after infection. Control mice received an equivalent volume of phosphate-buffered saline administered using the same procedure.

### Sample processing and cell isolation

Spleens were mechanically dissociated between sterile frosted glass slides (Fisher Scientific; #12-550-343) in RBC lysis buffer (Thermo Fisher Scientific; #00-4333-57), and the resulting single-cell suspensions were passed through a 40 µm strainer (Fisher Scientific; #22-363-547) as previously described.^41^ Splenocytes were resuspended in complete RPMI medium consisting of RPMI 1640 with L-glutamine and sodium bicarbonate (Sigma-Aldrich; #R8758), 10% fetal bovine serum (Thermo Fisher Scientific; #A2720801), and 1% penicillin/streptomycin (Sigma-Aldrich; #P4333). BM cells were flushed from femurs and tibiae into complete RPMI medium using a 30-gauge needle attached to a 10 mL syringe, as previously described.^42^ Human BM mononuclear cells were isolated from aspirates by Ficoll-Paque PREMIUM 1.074 (Cytiva; #17544652) density gradient centrifugation and washed in RPMI medium.

### In vitro cultures

Splenocytes were seeded at 1 × 10^6^ cells per well in a 96-well plate and incubated for 18 h at 37 °C in a humidified atmosphere with 5% CO_2_, in the presence of recombinant TGF-β (20 ng/mL), AMP (4 µg/mL), the A1 receptor antagonist PSB-36 (2.5 µg/mL), or the A2AR antagonist ZM241385 (500 ng/mL). Following incubation, cells were harvested, washed, and analyzed by flow cytometry according to our protocols.^41^^,^^43^ Where indicated, T cells were stimulated with soluble anti-CD3e (3 µg/mL; Waters Biosciences; #553057) and soluble anti-CD28 antibody (1 µg/mL; Waters Biosciences; #553294).

### Antibodies and flow cytometry analysis

Mouse cell-surface, intracellular, and intranuclear antigens were detected using the following antibodies: anti-TER119 (clone TER-119), anti-CD71 (RI7217), anti-CD45 (30-F11), anti-CD3 (145-2C11), anti-CD4 (GK1.5), anti-CD8 (53–6.7), anti-arginase-1 (A1exF5), anti-Latency Associated Peptide (LAP; TW7-16B4), anti-VISTA (MIH63), anti-CD39 (24DMS1), anti-CD73 (TY/23), anti-PD-L1 (10F.9G2), anti-TNF-α (MP6-XT22), and anti-pSMAD3 (O72-670). Human cell-surface antigens were detected using anti-CD235a (HIR2), anti-CD71 (M-A712), anti-CD45 (UCHL1), anti-CD39 (A1), and anti-CD73 (AD2). Dead cells were excluded using the Zombie Aqua Fixable Viability Kit (BioLegend; #423102). Apoptosis was assessed using the FITC Annexin V Apoptosis Detection Kit (BD Pharmingen; #556547) according to the manufacturer’s instructions, and ROS were measured using DCFDA (Abcam; #ab113851). For intracellular staining, cells were fixed and permeabilized using Cytofix/Cytoperm (Waters Biosciences; #554714), followed by staining with the appropriate antibody as previously described.^44–46^ For intranuclear staining, cells were permeabilized using nuclear permeabilization buffer (Thermo Fisher Scientific; #00-8333-56) and stained with anti-FOXP3 (FJK-16s) as previously reported.^47^ All stained cells were fixed in 4% paraformaldehyde prior to acquisition. Data were acquired on an LSRFortessa Cell Analyzer (Waters Biosciences) or a Cytek Aurora (Cytek Biosciences) and analyzed using FlowJo software (version 10.10; TreeStar).

### scRNA-seq library preparation

Splenocytes from 4T1 tumor-bearing mice were treated with RBC Lysis Buffer (Thermo Fisher Scientific; #00-4333-57) and pooled. CECs were enriched by positive selection using a TER119-PE antibody and anti-PE microbeads (Miltenyi Biotec; #130-048-801) followed by magnetic separation using LS columns (Miltenyi Biotec; #130-042-401). Enriched cells were subsequently subjected to fluorescence-activated cell sorting. Viable TER119^+^CD71^+^ cells were sorted into 3 populations based on CD11b and CD45 expression: CD11b^+^CD45^+^, CD11b^−^CD45^+^, and CD11b^−^CD45^−^. At least 50,000 live cells were collected for each population, and postsort purity and viability were confirmed. Cells were resuspended at a concentration of 1,000 cells/µL, and 10,000 cells per sample were loaded onto a 10x Genomics Chromium platform according to the manufacturer’s instructions. Libraries were generated using the Chromium Next GEM Single Cell 3′ Gene Expression v3.1 Dual Index kit (10x Genomics) and sequenced on a NovaSeq X Plus platform (Illumina).

### scRNA-seq data analysis

Sequencing reads were aligned and quantified against the 10x Genomics mm10-2020-A mouse reference genome (GRCm38) using Cell Ranger. Filtered gene expression matrices were used for downstream analysis. Quality control, clustering, and transcriptomic analysis were performed using Seurat v5.5.0, as previously described.^48^^,^^49^ Each sample was independently quality controlled by retaining genes expressed in at least 3 cells and cells expressing at least 200 genes with less than 5% mitochondrial transcript content. The 3 quality-controlled samples were merged into a single Seurat object for downstream analysis. Data were normalized and variance-stabilized using *SCTransform*, and the top 2,000 highly variable genes were identified using *FindVariableFeatures* with the variance-stabilizing transformation (vst) method. Principal component analysis was performed on variable genes, and 40 principal components were retained for downstream analyses. Cell clustering was performed using *FindNeighbors* and *FindClusters* at a resolution of 0.5, selected based on clustree analysis,^50^ and clusters were visualized using Uniform Manifold Approximation and Projection. For pathway analysis, genes were ranked by average log_2_ fold change from a Wilcoxon rank sum test using *FindMarkers*, comparing CD39^+^ and CD39^−^ cells. The ranked list was tested by gene set enrichment analysis^51^ using clusterProfiler^52^ against the mouse MSigDB hallmark, Gene Ontology biological process, and Reactome collections,^53^ with gene sets of 10 to 500 genes and a Benjamini-Hochberg–adjusted *P *< 0.05 threshold. Similar analyses were performed on publicly available scRNA-seq datasets GSE89754 (fetal liver) and GSE244806 (rodent malaria model).^54^^,^^55^

### ATP hydrolysis assay

CD39^+^ CECs, CD39^−^ CECs, and T cells were sorted as described previously and cultured in RPMI medium supplemented with 20 µM exogenous ATP (Thermo Fisher Scientific; #R0441) for 30 minutes. Culture supernatants were collected, and AMP levels were measured using an AMP assay kit (Abcam; #ab273275).

### T cell functional assays

T cells were isolated from BALB/c splenocytes using the EasySep Mouse T Cell Isolation Kit (STEMCELL Technologies; #19851) and labeled with CellTrace Violet (CTV) (Thermo Fisher Scientific; #C34571) to assess proliferation by dye dilution. Splenic CECs from 4T1 tumor-bearing mice were isolated in two steps: TER119^+^ cells were first enriched by magnetic selection using a TER119-PE antibody with anti-PE microbeads (Miltenyi Biotec; #130-048-801) on LS columns (Miltenyi Biotec; #130-042-401), and TER119^+^CD71^+^ cells were then sorted by fluorescence-activated cell sorting into CD39^+^ and CD39^−^ populations using a Sony MA900 cell sorter. To assess the impact of CECs on T cell proliferation, sorted CECs were cocultured with CTV-labeled T cells at the indicated ratios for 72 h, together with mitomycin C–treated splenocytes (CEC:splenocyte ratio, 1:10) as antigen-presenting cells and soluble anti-CD3 antibody (3 µg/mL) (Waters Biosciences; #553057) for T cell activation. Proliferation was quantified by flow cytometric analysis of CTV dilution, as previously reported.^56^

### Statistical analysis

Two-group comparisons were analyzed using an unpaired Student’s *t* test when data satisfied assumptions of normality and equal variance; when these assumptions were not met, the Mann-Whitney *U* test was used instead. For paired data, the Wilcoxon signed rank test was used. Comparisons across more than two groups were performed using 1-way analysis of variance, or the Kruskal-Wallis test with Dunn’s post hoc correction when parametric assumptions were violated. Data are reported as mean ± SEM, and statistical significance was defined as *P* < 0.05.

## Results

### CECs express CD39 in an age- and tumor-associated manner, with enrichment on the CD45^+^ subset

Given that CECs are highly abundant during the neonatal period in the spleen of mice,^13^^,^^57^ we first performed a temporal analysis of CD73 and CD39 expression across postnatal development. At every time point examined, a significantly greater proportion of CECs expressed CD39 than CD73 (Fig. 1A, B; Fig. S1A). Both ectoenzymes remained relatively stable during early development but exhibited a trend of increased expression at days 12 and 18 (Fig. 1A, B). We next compared their expression patterns across tissue compartments in neonatal, adult, and 4T1 tumor-bearing mice. In the spleen, CD39 expression was significantly higher in adult mice than in neonates, with the highest levels observed in 4T1 tumor-bearing mice, whereas CD73 expression showed no significant difference among the 3 groups. (Fig. 1C–E). It is worth mentioning that tumor-free adult mice have a very low frequency of splenic CECs, whereas CECs are highly enriched in 4T1 tumor-bearing mice (Fig. S1B). A similar pattern was observed in the BM, where both CD39 and CD73 expression were significantly elevated in CECs from 4T1 tumor-bearing mice compared with adults and neonates (Fig. 1C–E). Within the TME, CD39 was highly expressed by CECs, with a mean of approximately 30%, whereas CD73 expression remained low (Fig. 1D, E). These findings demonstrate that CD39 expression on splenic CECs is upregulated in 4T1 tumor-bearing mice, whereas CD73 was only increased on CECs from BM of tumor-bearing mice, suggesting a potential role for these molecules in the tumor setting.

**Figure 1:**
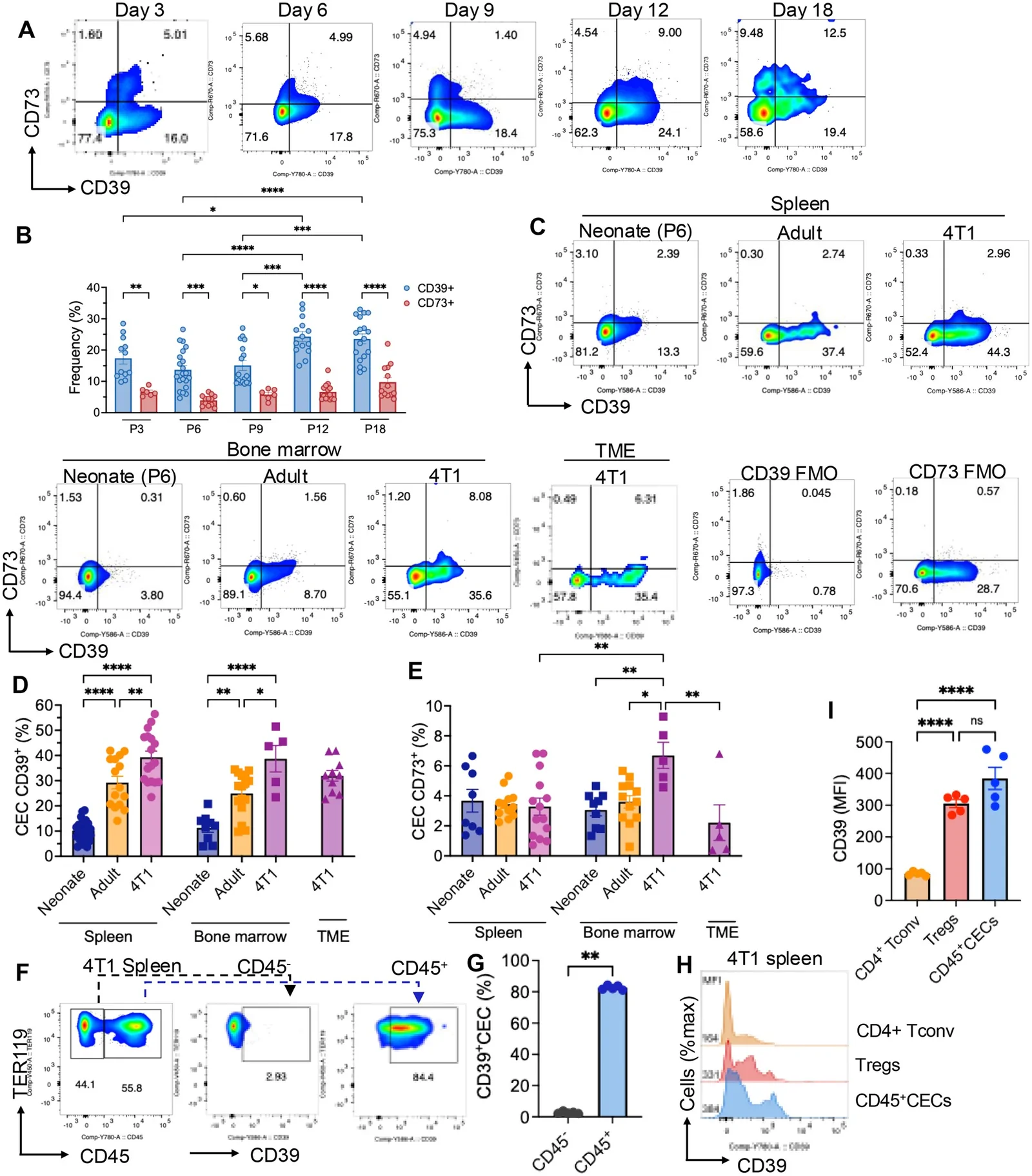
CD39 and CD73 expression on CECs across development, tissue compartments, and immune cell populations. (A) Representative flow cytometry plots and (B) cumulative frequencies of CD39^+^ and CD73^+^ CECs across postnatal days 3, 6, 9, 12, and 18. (C) Representative flow cytometry plots and cumulative frequencies of (D) CD39 and (E) CD73 expression on CECs in the spleen, BM, and TME of postnatal day 6, adults, and 4T1 tumor-bearing mice. (F) Representative flow cytometry plots and (G) cumulative CD39 abundance on CD45^−^ versus CD45^+^ CECs. (H) Representative histogram and (I) cumulative CD39 expression intensity on CD4^+^ T cells, Tregs, and CD45^+^ CECs. *P < 0.05, **P ≤ 0.01, ***P ≤ 0.001, and ****P ≤ 0.0001. FMO, fluorescence minus one; MFI, mean fluorescence intensity; Tconv, conventional T cell.

To determine whether CD39 is preferentially expressed by erythroid progenitors or precursors, we stratified CECs based on CD45 expression.^12^ Our analysis demonstrated that CD45^+^ CECs expressed CD39 at a significantly higher frequency than CD45^−^ CECs (Fig. 1F, G). We next evaluated the intensity of CD39 expression on CECs relative to two established CD39-expressing populations, conventional CD4^+^ T cells and Tregs. CD45^+^ CECs expressed CD39 at a significantly higher intensity than conventional CD4^+^ T cells and at levels comparable to Tregs in the spleen of 4T1 tumor-bearing mice (Fig. 1H, I). Together, these data identify CD39 as a consistent feature of CECs that is increased in the spleen and BM of adult and tumor-bearing mice, preferentially expressed by immature CD45^+^ cells, and maintained at levels comparable to those observed in Tregs.

### CECs expressing CD39 and CD73 are enriched during pregnancy and infection

Previous studies have implicated CECs in both pregnancy and infection.^15^^,^^17–19^ To determine whether physiological and pathological conditions beyond the tumor setting influence CD39 and CD73 expression, we examined the effects of pregnancy and infection on their expression in CECs. We found that pregnancy was associated with a significant increase in the frequency of splenic CECs (Fig. 2A, B). While only a small proportion of splenic CECs from both tumor-free and tumor-bearing mice express CD73 (Fig. 1E), pregnant mice exhibited a significantly higher frequency of CD39^+^CD73^+^ subsets among splenic CD45^+^ CECs (Fig. 2C, D). In both fetal liver and fetal gut, approximately 15% to 20% of CECs expressed CD39 and 10% to 15% expressed CD73 (Fig. 2C; Fig. S1C). We next assessed whether infection with *B. pertussis* affected CD39 and CD73 expression. In infected mice, CD73 expression was increased in CD45^−^ CECs, whereas both CD39 and CD73 expression were significantly elevated in the CD45^+^ CEC subset (Fig. 2E–G). To determine whether these observations extend to humans, we examined CD39 and CD73 expression on human BM-derived CECs. Flow cytometric analysis of samples revealed that a proportion of human CECs expressed CD39, with a subset coexpressing CD73 (Fig. 2H, I; Fig. S1D), supporting the relevance of these ectonucleotidases in human CECs. Collectively, these findings indicate that ectonucleotidase-expressing CECs are enriched under both physiological and pathological conditions.

**Figure 2:**
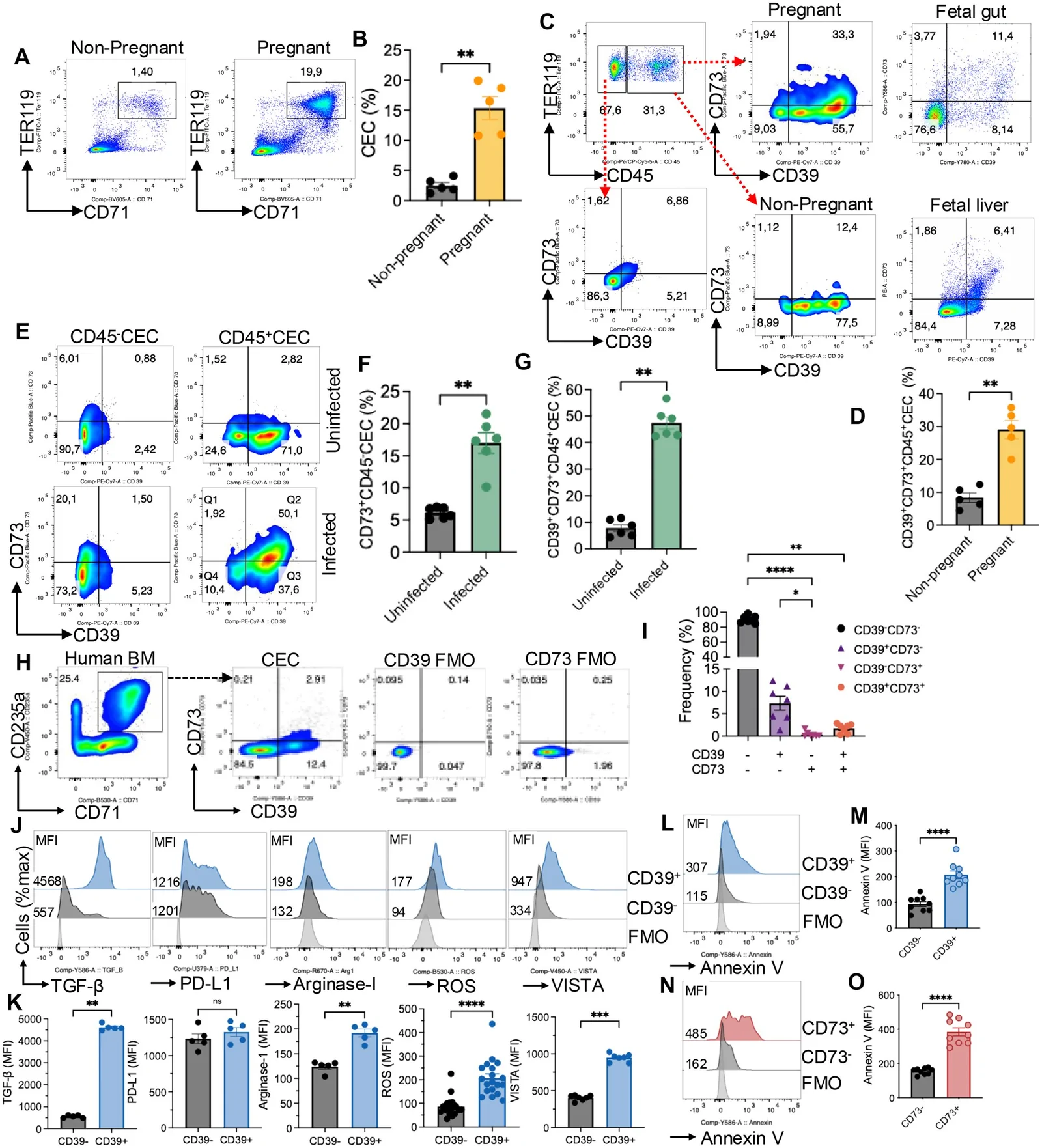
CD39 and CD73 expression on CECs across physiological and pathological states, human bone marrow, and their relationship to immunomodulatory molecules. (A) Representative flow cytometry plots and (B) cumulative frequencies of CECs in nonpregnant and pregnant mice. (C) Representative flow cytometry plots of CD39 and CD73 expression on CD45^−^ CECs in nonpregnant mice, CD45^+^ CECs in nonpregnant and pregnant mice, and fetal gut and fetal liver CECs. (D) cumulative frequencies of CD39^+^CD73^+^ CD45^+^ CECs. (E) Representative flow cytometry plots of CD39 and CD73 expression on CD45^−^ and CD45^+^ CECs in uninfected mice and mice infected with *B. pertussis*, with cumulative frequencies of (F) CD73^+^ CD45^−^ CECs and (G) CD39^+^CD73^+^CD45^+^ CECs. (H) Representative flow cytometry plots and (I) corresponding cumulative data of CD39 and CD73 expression on CECs in human bone marrow. (J) Representative histograms and (K) cumulative mean fluorescence intensity (MFI) of TGF-β, PD-L1, arginase-I, ROS, and VISTA on CD39^−^ and CD39^+^ CECs. (L) Representative histogram and (M) cumulative MFI of Annexin V on CD39^−^ and CD39^+^ CECs. (N) Representative histogram and (O) cumulative MFI of Annexin V on CD73^−^ and CD73^+^ CECs. *P < 0.05, **P ≤ 0.01, and ****P ≤ 0.0001. FMO, fluorescence minus one.

### CD39^+^ CECs represent a highly immunomodulatory CEC subset

To better understand the phenotypic properties of CD73^+^ and CD39^+^ CECs, we evaluated the expression of established immunomodulatory markers in these subsets.^24^^,^^26^^,^^28^ CD39^+^ CECs expressed significantly higher levels of TGF-β, arginase-I, ROS, and VISTA than CD39^−^ CECs, whereas PD-L1 expression remained unchanged (Fig. 2J, K). Notably, both CD39^+^ CECs and CD73^+^ CECs also exhibited increased susceptibility to apoptosis (Fig. 2L–O), suggesting that, despite expressing potent immunomodulatory mediators, these cells may be relatively short-lived. Having observed that CD39^+^ CECs expressed elevated of TGF-β, and given that CECs are an established source of this cytokine,^12^^,^^26^ we next investigated whether TGF-β regulates ectonucleotidase expression in these cells. CECs were cultured with recombinant TGF-β (rTGF-β) and surface expression of CD39 and CD73 was assessed. Treatment with rTGF-β significantly increased the expression of both CD39 (Fig. S2A, B) and CD73 (Fig. S2C, D) on CECs. To determine whether canonical TGF-β signaling was activated, we measured intracellular phospho-SMAD3 (p-SMAD3), which was significantly increased in rTGF-β–treated CECs (Fig. S2E, F). Consistent with this finding, CD39^+^ CECs exhibited higher intracellular p-SMAD3 levels than their CD39^−^ counterparts (Fig. S2G, H). Together, these findings indicate that TGF-β promotes CD39 and CD73 expression on CECs through activation of the SMAD-3 signaling pathway. Although TGF-β treatment increased CD39 and CD73 expression alongside SMAD3 phosphorylation in CECs, we did not inhibit or genetically perturb SMAD3. We therefore cannot determine whether SMAD3 is required for TGF-β–induced ectonucleotidase upregulation; other downstream pathways may contribute.

### scRNA-seq reveals that CD39^+^ CECs exhibit a distinct adenosine-generating and immunomodulatory transcriptional program

To characterize ectonucleotidase expression and the transcriptional programs associated with CD39 in CECs, we performed scRNA-seq on splenic CECs isolated from 4T1 tumor-bearing mice. We profiled CD45^−^ CECs alongside CD45^+^ CECs, the latter further subdivided into erythroid-myeloid (CD11b^+^CD45^+^) and canonical (CD11b^−^CD45^+^) progenitors. CD45^−^ and CD45^+^ CECs formed distinct populations in Uniform Manifold Approximation and Projection space (Fig. 3A), and unsupervised clustering identified 14 CEC clusters (Fig. 3B). Consistent with our flow cytometric analysis, *Entpd1* (CD39) and *Nt5e* (CD73) expression was largely restricted to CD45^+^ CECs (Fig. 3C). Within this compartment, *Entpd1* was broadly expressed, whereas *Nt5e* was detected in only a minority of cells, consistent with the relatively small CD39^+^CD73^+^ population identified by flow cytometry. *Entpd3* (NTPDase3), another ectonucleotidase family member, was predominantly expressed in the CD45^+^CD11b^+^ subset (Fig. 3D), suggesting that CD39 is not the only NTPDase available to these cells.

**Figure 3:**
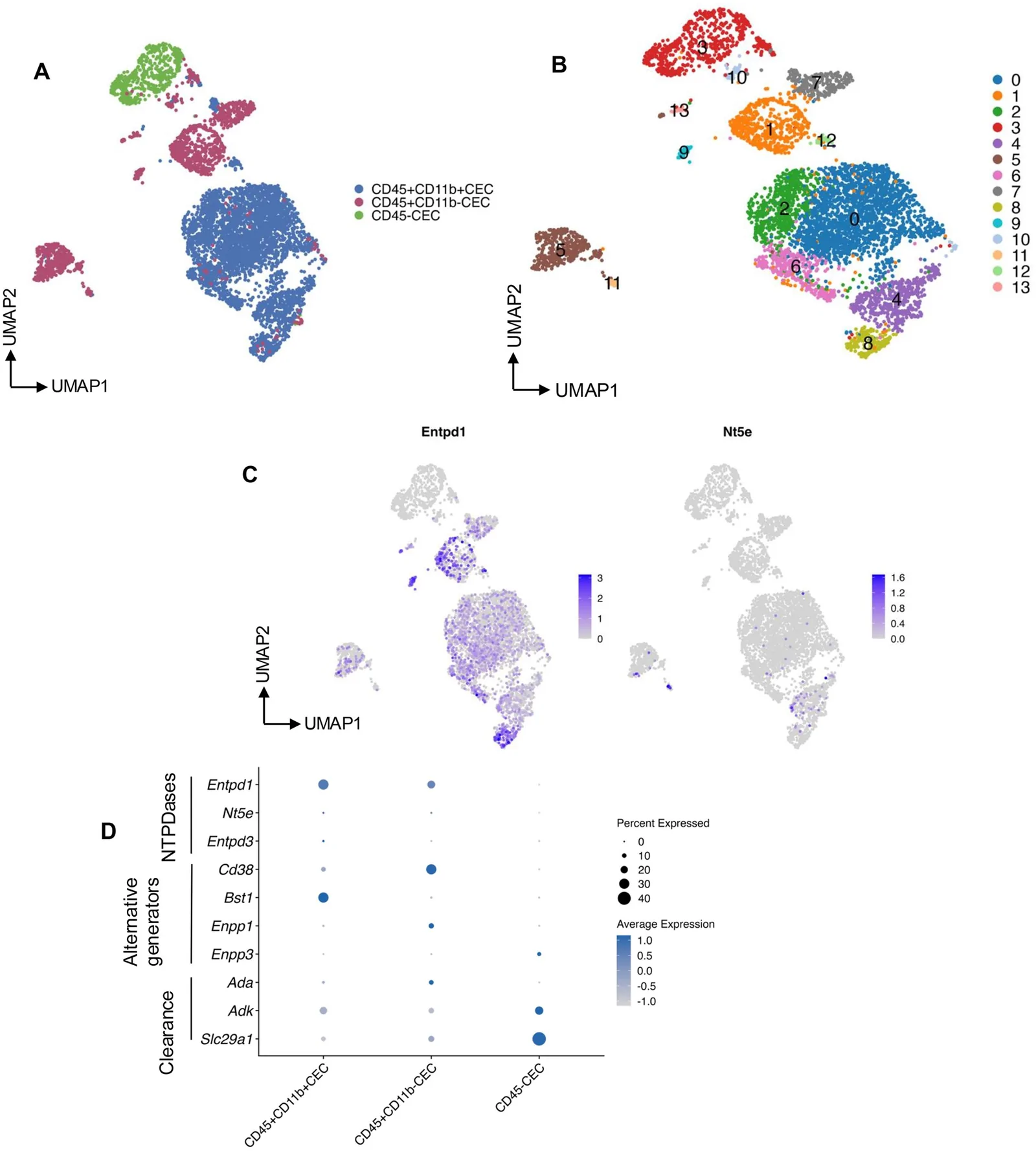
Single-cell transcriptomic profiling reveals higher expression of CD39 gene (*Entpd1*) in CD45^+^ CECs. (A) Unsupervised graph-based clustering and Uniform Manifold Approximation and Projection (UMAP) visualization of 3 CEC subsets: CD45^−^ CECs, CD45^+^CD11b^+^ CECs, and CD45^+^CD11b^−^ CECs. (B) UMAP of 14 Seurat clusters identified across the merged CEC subsets. (C) Feature plots showing scaled expression of *Entpd1* and *Nt5e* in CECs. (D) Dot plot showing expression of NTPDase family genes and genes involved in alternative AMP generation and clearance in CECs.

We next examined the expression of CD39-independent enzymes involved in adenosine metabolism. Among alternative adenosine-generating enzymes, *Cd38*, *Bst1*, and *Enpp1* were highly expressed in CD45^+^ CECs, whereas *Enpp3* was enriched in CD45^−^ CECs. In contrast, transcripts associated with adenosine clearance, including *Adk* and *Slc29a1*, were most abundant in CD45^−^ CECs, while *Ada* expression was enriched in the CD11b^−^CD45^+^ subset (Fig. 3D). These findings indicate that CECs possess multiple pathways for adenosine production beyond the canonical CD39/CD73 axis and suggest that adenosine metabolism is partitioned among CEC subsets, with adenosine-generating pathways predominating in CD45^+^ CECs and adenosine clearance mechanisms enriched in CD45^−^ CECs.

To directly associate these transcriptional programs with CD39 expression, we stratified CECs according to *Entpd1* (CD39) expression (Fig. 4A). Approximately 26.7% of CECs expressed *Entpd1* (CD39^+^), whereas the remaining cells lacked detectable *Entpd1* expression (Fig. 4B). CD39^+^ CECs exhibited increased expression of *Entpd3* together with the alternative adenosine-generating enzymes *Cd38* and *Bst1*, whereas CD39^−^ CECs preferentially expressed the adenosine clearance genes *Adk* and *Slc29a1* (Fig. 4C), reinforcing the distinction between adenosine-generating and adenosine-clearing CEC subsets.

**Figure 4:**
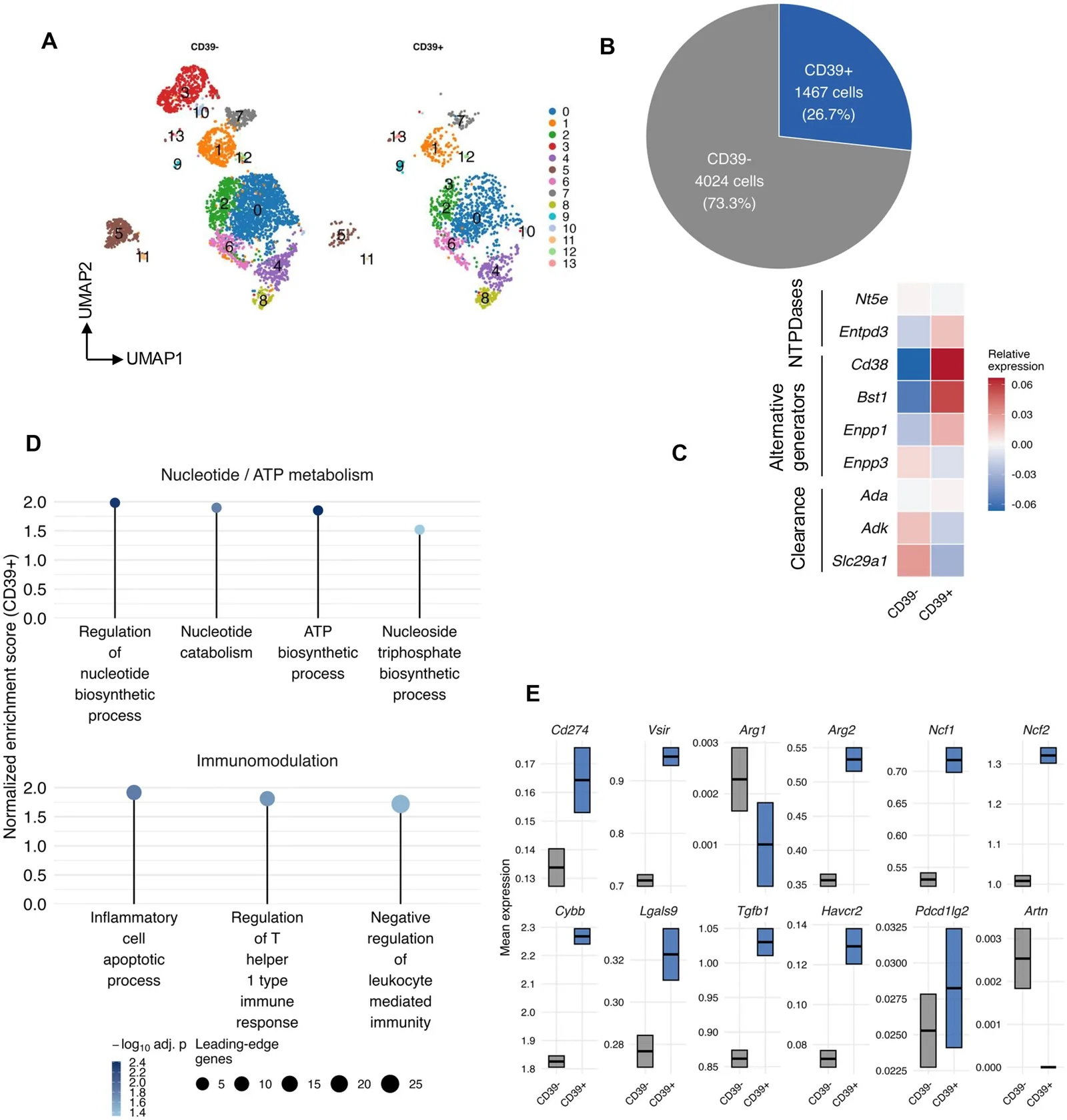
Single-cell transcriptomic profiling reveals nucleotide metabolism and immunomodulatory gene expression in CECs. (A) UMAP of CD39^−^ and CD39^+^ CECs, with Seurat clusters shown for each population. (B) Proportion of CD39^+^ CECs and CD39^−^ CECs. (C) Heatmap of genes associated with NTPDases, alternative AMP generation, and AMP-clearing enzymes in CD39^−^ versus CD39^+^ CECs. (D) Pathway analysis of nucleotide metabolism– and immunomodulation-associated genes enriched in CD39^+^ CECs. (E) Box plots comparing expression of immunomodulatory mediator genes in CD39^−^ versus CD39^+^ CECs.

Pathway analysis of genes differentially expressed between CD39^+^ and CD39^−^ CECs demonstrated enrichment of nucleotide biosynthesis, nucleotide catabolism, and nucleoside triphosphate biosynthetic processes in CD39^+^ CECs (Fig. 4D), consistent with an active role in purine nucleotide metabolism. CD39^+^ CECs were also enriched for immune-related pathways, including inflammatory cell apoptosis, regulation of the Th1 response, and negative regulation of leukocyte-mediated immunity, suggesting that these cells couple nucleotide metabolism with immunoregulatory functions.

Finally, we examined the expression of immunomodulatory genes. Relative to CD39^−^ CECs, CD39^+^ CECs expressed higher levels of *Cd274* (PD-L1), *Vsir* (VISTA), *Arg2* (arginase-II), the NADPH oxidase components *Ncf1*, *Ncf2*, and *Cybb* (associated with ROS generation), *Lgals9* (Gal-9), *Tgfb1* (TGF-β), *Havcr2* (Tim-3), and *Pdcd1lg2* (PD-L2). In contrast, CD39^−^ CECs showed higher expression of *Arg1* (arginase-I) and *Artn* (Artemin) (Fig. 4E). To determine whether CD39^+^ CECs exhibit similar transcriptional profiles across different contexts, we analyzed publicly available scRNA-seq datasets from mouse fetal liver and spleens from a rodent malaria infection model.^54^^,^^55^ CECs were the most dominant cell population in the fetal liver (Fig. S3A), and a subset expressed *Entpd1* and *Ptprc* genes (Fig. S3B, C). However, we observed that fetal liver CD39^+^ CECs differed transcriptionally from their tumor-induced counterparts, exhibiting lower expression of *Arg1*, *Cd274*, *Pdcd1Ig2*, *Ncf1*, and *Ncf2* (Fig. S3D). We extended our analysis to the rodent malaria model, we identified a CEC subset population, a small subset of which expressed *Entpdi* and *Ptprc* (Fig. S4A, B). Compared with CD39^−^ CECs, CD39^+^ CECs exhibited higher expression of ROS-associated genes (*Cybb*, *Ncf1*, and *Ncf2*), as well as *Lgals3* and *Cd274*, and lower expression of *Tgfb1,* with no detectable differences in *Arg1* and *Arg2* expression (Fig. S4C). These analyses reveal context-dependent differences in the transcriptional profiles of CD39^+^ CECs across physiological and pathological conditions. Finally, to explore potential factors associated with the increased susceptibility of CD39^+^ CECs to apoptosis (Fig. 2L, M), we examined *P2rx7*, which encodes the ATP-gated purinergic receptor P2X7. CD39^+^ CECs exhibited higher *P2rx7* expression than CD39^−^ CECs (Fig. S4D). Although further studies are required to establish the mechanism, this suggests a potential link between purinergic signaling and their increased susceptibility to apoptosis.^58^

Collectively, these findings indicate that CD39^+^ CECs exhibit a transcriptional program consistent with enhanced nucleotide metabolism, including expression of CD39 and other enzymes involved in adenosine generation. However, their broader transcriptional profiles vary across biological contexts, indicating that their functional properties should not be assumed to be uniform.

### CD39^+^ CECs are enzymatically active and suppress T cell proliferation in part through A2AR signaling

To determine whether CD39 expressed on splenic CECs from breast cancer-bearing mice is enzymatically active, we performed an ATP hydrolysis assay. CD39^+^ and CD39^−^ CECs were isolated from the spleens of 4T1 tumor-bearing mice, with T cells included as a control, and cultured in the presence of exogenous ATP. CD39 activity was assessed by measuring AMP accumulation in the culture supernatant (Fig. 5A). AMP concentrations were significantly higher in supernatants from CD39^+^ CECs than in those from CD39^−^ CECs or T cells (Fig. 5B), indicating that CD39^+^ CECs actively hydrolyze extracellular ATP to AMP.

**Figure 5:**
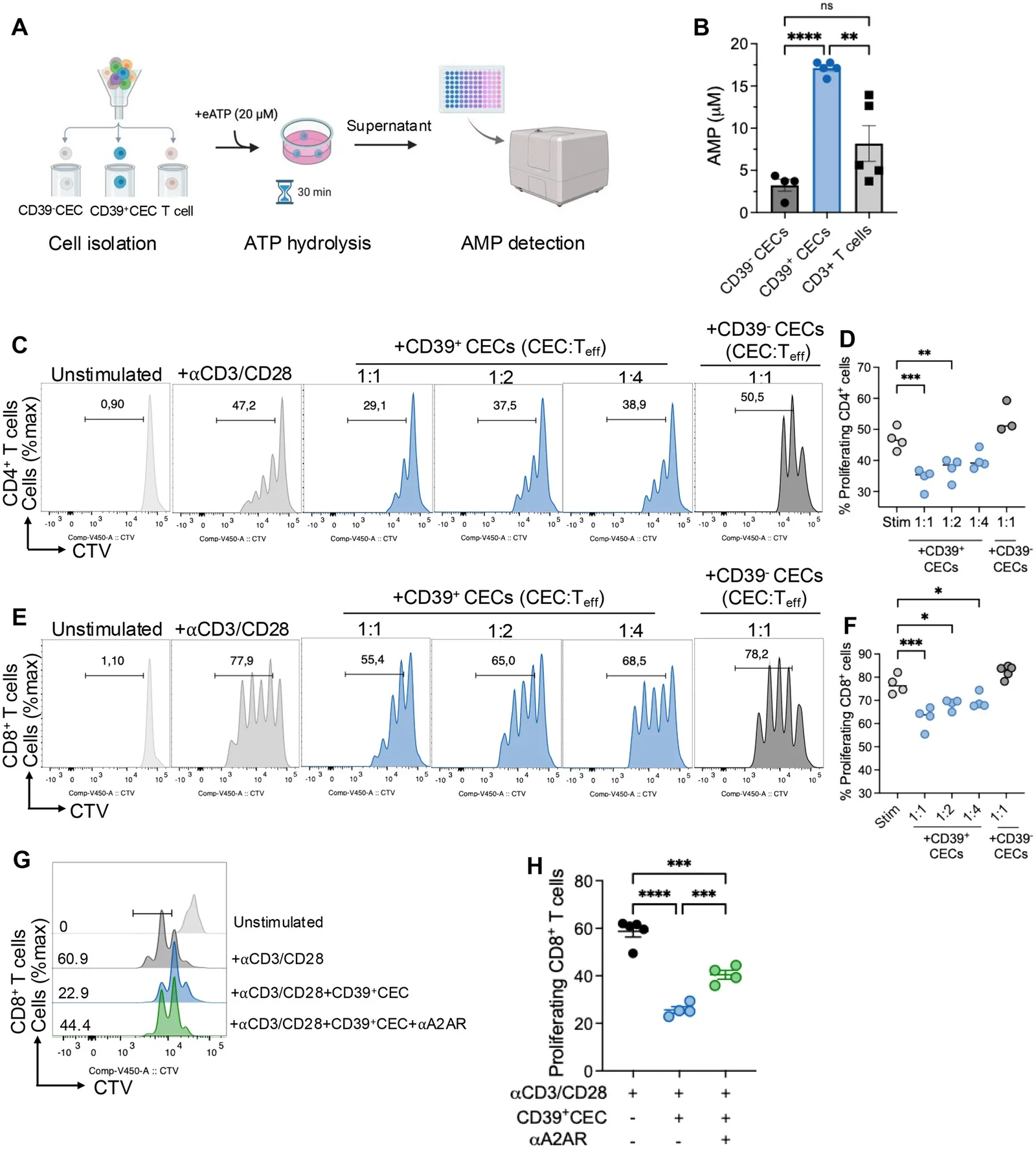
CD39^+^ CECs hydrolyze ATP to AMP and suppress T cell proliferation in an adenosine receptor–dependent manner. (A) Schematic of the ATP hydrolysis assay workflow: splenic CECs and T cells were isolated from 4T1 tumor-bearing mice, cultured with exogenous ATP (eATP) for 30 minutes, and AMP was measured in the culture supernatant. (B) AMP concentration in culture supernatant from CD39^−^ CECs, CD39^+^ CECs, and CD3^+^ T cells. (C) Representative histograms and (D) cumulative proliferation of activated CD4^+^ T cells cultured alone or with CD39^−^ or CD39^+^ CECs. (E) Representative histograms and (F) cumulative proliferation of activated CD8^+^ T cells cultured alone or with CD39^−^ or CD39^+^ CECs. (G) Representative histogram and (H) cumulative proliferation of activated CD8^+^ T cells cultured alone or with CD39^+^ CECs, in the presence or absence of the adenosine A2A receptor antagonist ZM241385. *P < 0.05, **P ≤ 0.01, ***P ≤ 0.001, and ****P ≤ 0.0001. ns, not significant.

Because adenosine generated downstream of CD39 is a well-established immunosuppressive mediator,^34^ we next examined whether CD39^+^ CECs suppress T cell responses. CD3/CD28-activated T cells were cocultured with CD39^+^ or CD39^−^ CECs isolated from the spleens of 4T1 tumor-bearing mice, and T cell proliferation was assessed. Proliferation of both CD4^+^ (Fig. 5C, D) and CD8^+^ (Fig. 5E, F) T cells was significantly reduced in the presence of CD39^+^ CECs but not CD39^−^ CECs. This suppressive effect was dose dependent, with T cell proliferation progressively increasing as the CEC cell ratio decreased, indicating that suppression correlated with the abundance of CD39^+^ CECs. Given the low expression of CD73 on CECs, we hypothesized that T cell–expressed CD73 might cooperate in trans with CEC-expressed CD39 to convert CEC-generated AMP into adenosine. Consistent with this possibility, a higher proportion of splenic CD4^+^ T cells expressed CD73, and at greater intensity, than CD8^+^ T cells (Fig. S4E, F). To determine whether adenosine signaling contributed to this suppressive activity, rather than other immunomodulatory mediators expressed by CD39^+^ CECs, we added the selective A2AR antagonist ZM241385 to CD39^+^ CEC and T cell cocultures. Pharmacological blockade of A2AR partially but significantly restored CD8^+^ T cell proliferation (Fig. 5G, H), indicating that adenosine signaling through A2AR contributes to, but does not fully account for, the suppressive activity of CD39^+^ CECs. Together, these findings demonstrate that CD39^+^ CECs possess functional ectonucleotidase activity, hydrolyzing extracellular ATP to AMP, and suppress T cell proliferation in part through A2AR-dependent adenosine signaling.

### AMP suppresses T cell effector function through adenosine receptor signaling

Having shown that CD39^+^ CECs hydrolyze ATP to AMP and suppress T cell proliferation, we next investigated whether exogenous AMP also impairs T cell cytokine production. Whereas proliferation was assessed over 3 d with CECs remaining in coculture, the cytokine assay examined TNF-α production following a shorter exposure to exogenous AMP, providing a complementary readout of T cell effector function. CD3/CD28-activated CD4^+^ and CD8^+^ T cells were cultured in the presence of AMP, which significantly reduced TNF-α production by both CD4^+^ (Fig. 6A, B) and CD8^+^ (Fig. 6C, D) T cells. These findings indicate that the effects of exogenous AMP extend beyond inhibition of proliferation to reduced cytokine production.

**Figure 6:**
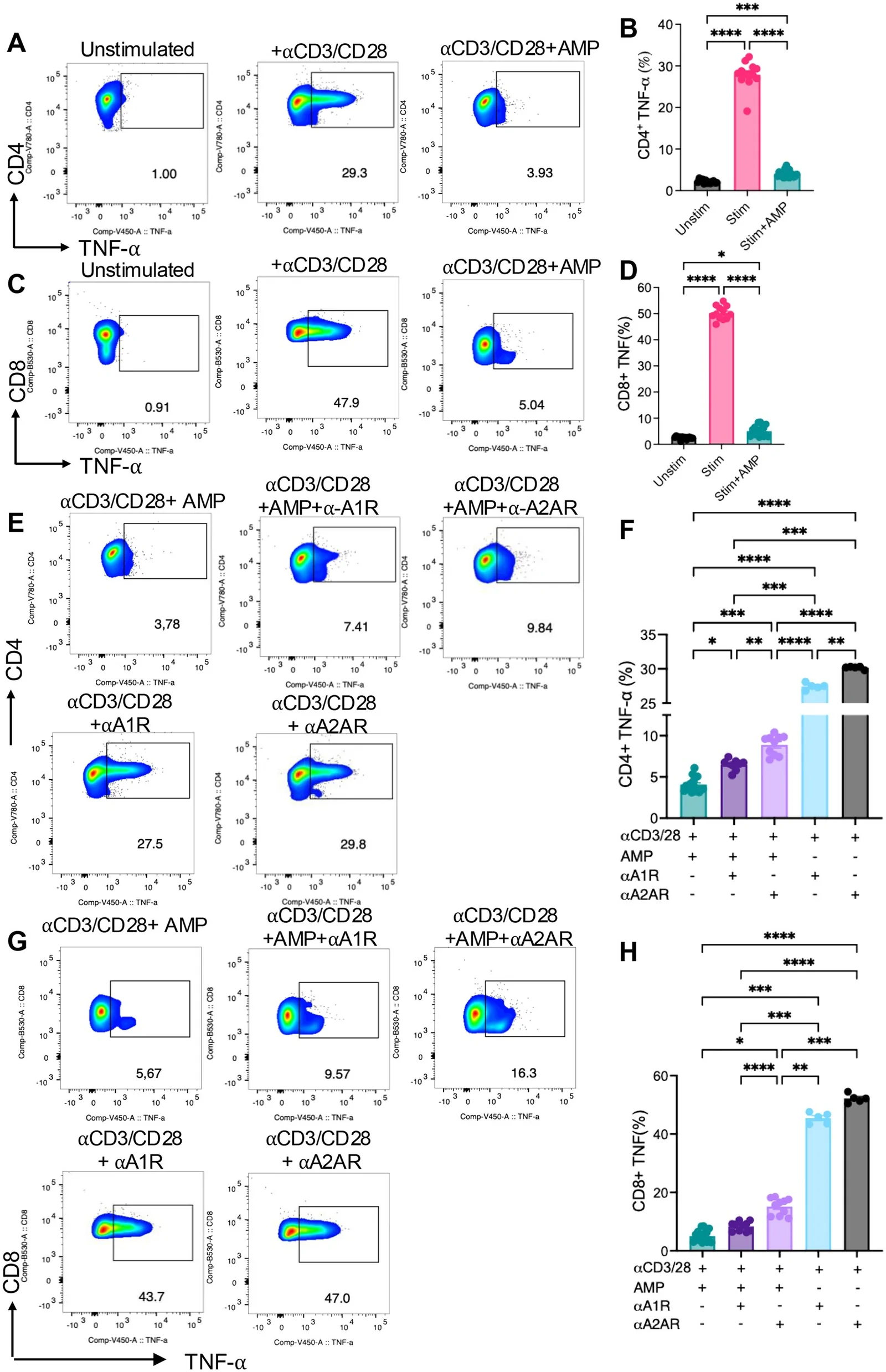
AMP suppresses T cell TNF-α production via adenosine A1 receptor (A1R) and A2AR signaling. (A) Representative flow cytometry plots and (B) cumulative frequencies of TNF-α^+^ CD4^+^ T cells that were unstimulated, stimulated with anti-CD3/CD28, or stimulated with anti-CD3/CD28 plus AMP. (C) Representative flow cytometry plots and (D) cumulative frequencies of TNF-α^+^ CD8^+^ T cells under the same 3 conditions. (E) Representative flow cytometry plots and (F) cumulative frequencies of TNF-α^+^ CD4^+^ T cells activated with anti-CD3/CD28 in the presence of AMP alone, AMP plus an A1AR antagonist (PSB-36), AMP plus an A2AR antagonist (ZM241385), or each antagonist alone as a control. (G) Representative flow cytometry plots and (H) cumulative frequencies of TNF-α^+^ CD8^+^ T cells under the same conditions as in panels E and F. *P < 0.05, **P ≤ 0.01, ***P ≤ 0.001, and ****P ≤ 0.0001.

To determine which adenosine receptors mediate this effect, we added selective antagonists of A1R and A2AR. In CD4^+^ T cells, blockade of either A1R or A2AR partially restored TNF-α production (Fig. 6E, F), whereas in CD8^+^ T cells, only A2AR blockade was effective (Fig. 6G, H). These findings indicate that AMP suppresses T cell effector function through adenosine receptor signaling, with CD4^+^ and CD8^+^ T cells exhibiting distinct receptor dependencies. Together, these results link the enzymatic activity of CD39^+^ CECs to a defined adenosine receptor–dependent mechanism of T cell immunosuppression.

## Discussion

Here, we investigated whether CECs, which expand in tumor-bearing hosts and suppress T cell responses, contribute to immunosuppression through the adenosinergic pathway. We found that CD39 was consistently expressed by CECs and was enriched in the spleen and BM of 4T1 tumor-bearing mice. Its expression was concentrated on immature CD45^+^ CECs at levels comparable to Tregs, whereas CD73 expression remained low. CD39 expression on CECs was also observed during pregnancy, in fetal liver/gut, and infection, as well as in human BM. scRNA-seq identified the CEC subsets expressing *Entpd1* and *Nt5e*, revealed additional enzymes potentially involved in extracellular adenosine metabolism, and showed that CD39^+^ CECs exhibited a broad immunomodulatory transcriptional program. Functionally, CEC-associated CD39 was enzymatically active and hydrolyzed extracellular ATP to AMP. CD39^+^ CECs suppressed CD4^+^ and CD8^+^ T cell proliferation, an effect partially reversed by A2AR blockade. Moreover, exogenous AMP impaired T cell effector function through adenosine receptor signaling, suggesting its extracellular conversion to adenosine.

Although our functional studies focused on TNBC, ectonucleotidase-expressing CECs were not restricted to the tumor setting. CD39^+^ CECs were abundant in the neonate and were further enriched during pregnancy and *B. pertussis* infection. CD39-expressing CECs, including a subset coexpressing CD73, were also detectable in human BM. This distribution parallels the physiological and pathological contexts in which CECs exert immunoregulatory functions, including the fetomaternal interface, early life, and infection,^11^^,^^14^^,^^15^^,^^18^^,^^19^ suggesting that CD39-mediated purine metabolism may be a recuring component of CEC-mediated immune regulation. In physiological settings such as pregnancy and the neonatal period, CEC-mediated immunosuppression limits excessive inflammation and promotes tolerance.^11^^,^^15^ The enrichment of CD39-expressing CECs, together with CD73 coexpression in selected subsets, suggests that adenosine signaling may contribute to this homeostatic and tolerogenic program. Our findings support a model in which TNBC coopts this pre-existing CEC immunoregulatory axis, redirecting a mechanism that normally supports developmental and fetomaternal tolerance toward the suppression of antitumor immunity.

Although CECs exhibited abundant CD39 expression, CD73 was detected on only a small proportion of cells across the compartments examined, consistent with the scRNA-seq finding that *Nt5e* expression was restricted to a minority of CD45^+^ CECs. Because CD39 and CD73 act sequentially, with CD73 catalyzing the conversion of AMP to adenosine,^31^ this asymmetric expression positions CECs primarily at the AMP-generating step of the adenosinergic pathway. CEC-derived AMP could subsequently be converted to adenosine in trans by CD73 expressed within the breast TME by tumor cells, Tregs, and myeloid populations,^30^^,^^37^^,^^59^ or through alternative ecto-5′-nucleotidases and alkaline phosphatases.^60^ This potential division of enzymatic labor distinguishes CECs from cells that coexpress CD39 and CD73, such as Tregs, and positions CECs as initiators of the adenosinergic cascade by supplying AMP to a CD73-rich microenvironment. Although extracellular AMP is generally thought to act following its dephosphorylation to adenosine, some studies have reported that AMP can activate adenosine receptors and induce downstream signaling without prior extracellular conversion.^61^^,^^62^ Thus, both CD73-dependent conversion of AMP to adenosine and, potentially, direct AMP-mediated signaling may contribute to the suppressive effects observed in our system. Consistent with this model, exogenous AMP suppressed T cell effector function, and this effect was partially reversed by adenosine receptor antagonists, supporting the involvement of adenosine receptor signaling. Given the low CD73 expression on CECs, CD73 on neighboring cells, including T cells, may cooperate in trans with CEC-expressed CD39 to convert CEC-generated AMP into adenosine. However, whether AMP-associated suppression reflects direct receptor engagement or extracellular conversion to adenosine remains to be determined.

CD39^+^ CECs suppressed T cell proliferation, whereas CD39^−^ CECs did not. A2AR blockade only partially restored proliferation, indicating that additional CD39-independent mechanisms contribute to this suppression. Consistent with this, CD39^+^ CECs coexpressed multiple immunomodulatory mediators at the protein or transcript level, including TGF-β, arginase-II, VISTA, Gal-9, TIM-3, PD-L2, and ROS, which are reported to contribute to CEC-mediated immunosuppression in different models.^15^^,^^22^^,^^57^^,^^63^ Thus, CD39-mediated purine metabolism operates within a broader suppressive program, suggesting that effective reversal of CEC-mediated immunosuppression may require targeting complementary pathways.

TGF-β is an established inducer of the CD39/CD73 axis across multiple immune lineages, driving ectonucleotidase expression on tumor-associated myeloid cells, effector T cells, and Tregs.^37^^,^^64–66^ We found that this relationship extends to CECs: recombinant TGF-β increased the expression of both CD39 and CD73 on CECs and activated canonical SMAD3. Consistent with an association between this pathway and the suppressive phenotype, CD39^+^ CECs exhibited higher baseline p-SMAD3 than their CD39^−^ counterparts. Because CECs are themselves a well-documented source of TGF-β,^12^^,^^13^^,^^26^ these observations raise the possibility of an autocrine or paracrine circuit in which CEC-derived TGF-β reinforces ectonucleotidase expression on the same or neighboring cells. Such a self-sustaining loop could sustain the suppressive phenotype within the TME, analogous to the TGF-β–driven regulatory circuits described in Tregs and MDSCs.^37^^,^^65^ Notably, CD39^+^ CECs exhibited greater immunosuppressive function despite increased apoptosis. This raises the possibility that dying CECs release ATP, which is hydrolyzed to AMP by CEC-expressed CD39 and subsequently converted to adenosine by CD73 on neighboring cells, including T cells as described for apoptotic tumor-associated Tregs.^67^ However, we did not directly measure apoptosis-associated ATP release or adenosine production in these cocultures and therefore cannot establish whether this pathway contributes to CEC-mediated suppression.

Although our convergent phenotypic, transcriptional, and functional analyses established CD39 and CD73 expression on CECs, and demonstrate the enzymatic activity of CEC-associated CD39, these findings should be interpreted considering several limitations. First, because CEC-specific CD39 loss-of-function experiments were not performed, we cannot determine whether CD39 is required for CEC-mediated T cell suppression or whether CD39 also identifies a CEC subset with other suppressive properties. Although our findings support a role for CD39-associated ATP-to-AMP metabolism, they do not establish the cell-autonomous contribution of CEC-expressed CD39 to immune suppression. Second, although CD39^+^ CECs hydrolyze extracellular ATP to AMP and exogenous AMP suppresses T cell effector function, we did not directly demonstrate that AMP generated by CEC-expressed CD39 mediates this suppression. Experiments that selectively disrupt CD39 activity in CECs and measure both AMP production and T cell responses will be needed to establish this causal link. Third, although we assessed AMP generation, we did not quantify adenosine in the suppression cultures. We therefore cannot distinguish direct effects of AMP from suppression mediated by its conversion to adenosine.

Fourth, our functional experiments were conducted using 4T1 tumor-bearing mice. Although this is a well-established and clinically relevant model of TNBC, tumor models vary in the extent of extramedullary hematopoiesis and immune composition, both of which could influence CEC expansion and phenotype. Confirmation in additional tumor models and patient samples will therefore be necessary to establish the generalizability of CD39^+^ CEC-mediated suppression. Fifth, although we demonstrate that CD39^+^ CECs hydrolyze ATP to AMP and that AMP suppresses T cell function through adenosine receptor signaling, the downstream events linking CEC-derived AMP to receptor engagement within the TME in vivo remain undefined. Determining where and how this suppressive pathway is completed within intact tumors will require in situ investigation. Sixth, our functional and transcriptional characterization of CD39^+^ CECs primarily relied on splenic cells from tumor-bearing mice, as the spleen is a major site of extramedullary erythropoiesis. Although we confirmed the presence of CD39^+^ CECs within the TME, whether their enzymatic and suppressive activities are equivalent to those of their splenic counterparts remains to be established. Building on these findings, future research should determine whether pharmacological inhibition of CD39, alone or in combination with blockade of complementary suppressive pathways, restores antitumor immunity in vivo. Additionally, determining whether CD39^+^ CECs have prognostic value or represent viable therapeutic targets will help define the clinical relevance of CEC-associated adenosinergic signaling in breast cancer. It is worth nothing that comparative transcriptomic analyses revealed distinct gene expression profiles in CD39^+^ CECs from TNBC, malaria infection, and fetal liver, suggesting that their phenotypes and functional properties may vary across physiological and pathological contexts.

In summary, we identify CD39 as a marker of CECs with enhanced immunosuppressive function and implicate purine metabolism as an additional pathway contributing to their immunoregulatory properties in TNBC. By demonstrating that CD39^+^ CECs hydrolyze extracellular ATP to AMP, while exogenous AMP promotes adenosine receptor–dependent suppression of T cell function, our findings position the adenosinergic axis alongside the previously described suppressive pathways of CECs and reveal a broader mechanism through which these cells regulate immune responses across physiological and pathological settings.

## Supplementary Material

vkag301_Supplementary_Data

## Data Availability

All data supporting the findings of this study are included in the main text and supplementary materials. Raw sequencing reads have been deposited in the National Center for Biotechnology Information Sequence Read Archive under BioProject accession PRJNA1263571. Processed data and gene-expression matrices are available in the National Center for Biotechnology Information Gene Expression Omnibus under accession GSE314744. scRNA-seq datasets related to fetal liver (GSE89754) and rodent malaria infection model (GSE244806) are publicly available. Additional data are available from the corresponding author, SE, upon reasonable request.
