# Supplementary material for: CD39+CD71+ erythroid cells exhibit enhanced purine metabolism and T cell–suppressive function in triple-negative breast cancer and other immunoregulatory settings
Source: J Immunol. 2026 Nov 13;215(11):vkag301. doi: 10.1093/jimmun/vkag301 (PMC13760227; doi:10.1093/jimmun/vkag301)
Supplement: Supplementary file 1 [file vkag301_supplementary_data.pdf]

S Fig. 1

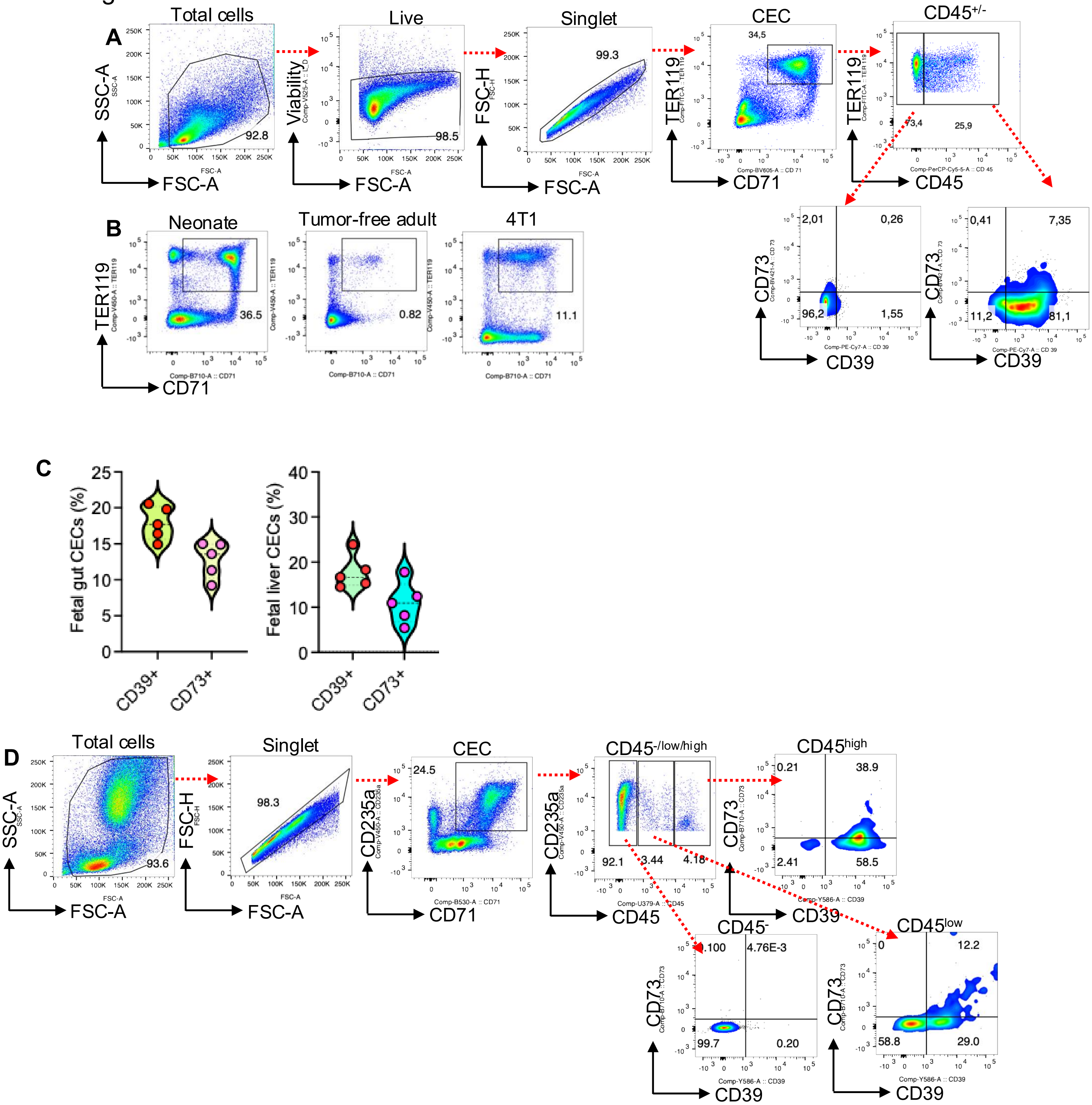

S Fig 1. (A) Representative gating strategy for CD39 and CD73 expression on splenic CECs from neonatal mice. **(B)** Representative flow cytometry plots showing CEC frequency in tumor-free neonate, tumor-free adult, and 4T1 tumor-bearing mice. **(C)** Cumulative data showing the frequency of CD39 and CD73 expressing cells among mouse fetal gut and fetal liver CECs. **(D)** Representative gating strategy for CD39 and CD73 expression on CECs from human bone marrow.

S Fig. 2

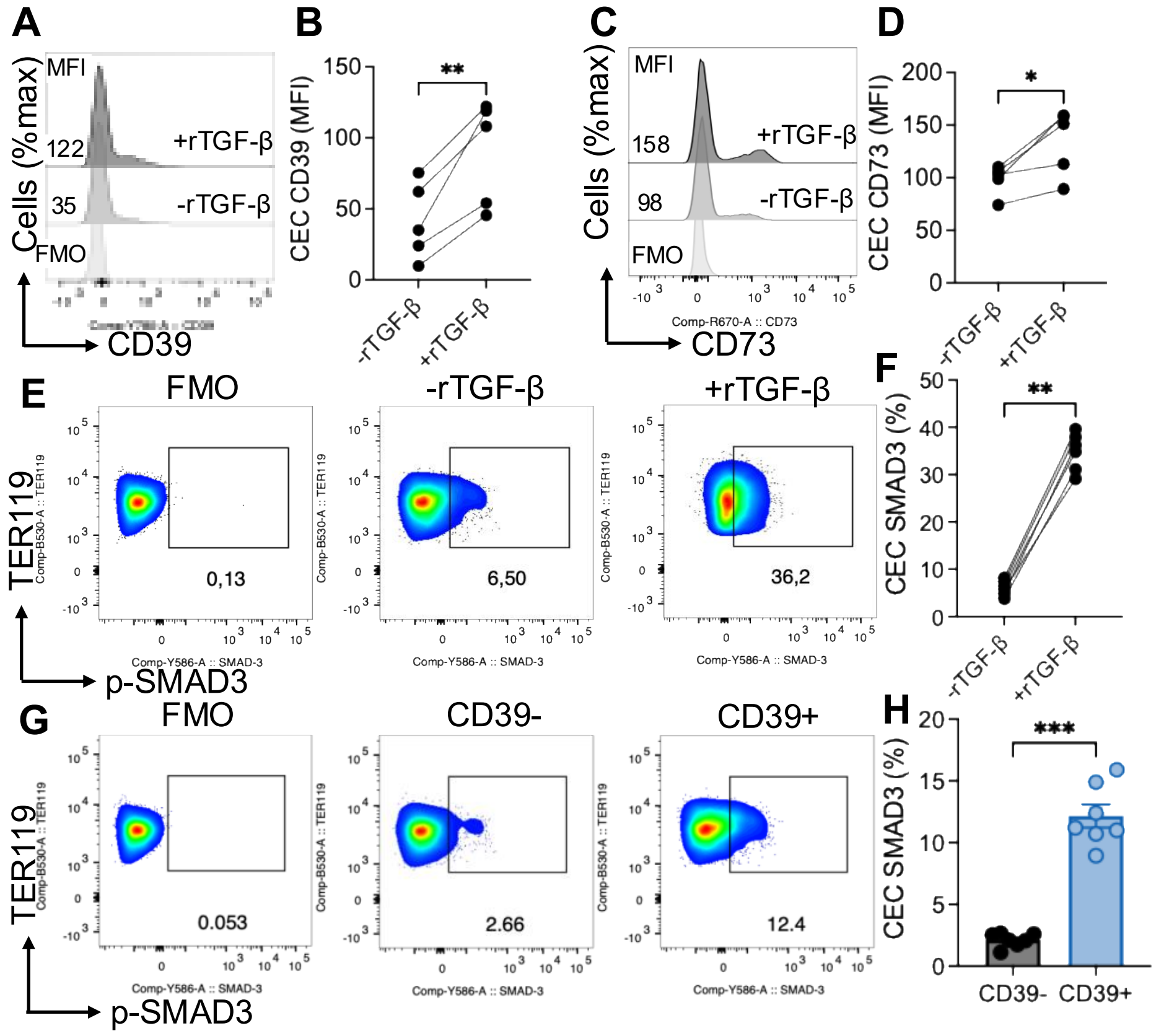

S Fig 2. (A) Representative histogram and **(B)** cumulative data for CD39 expression intensity on splenic CECs untreated or treated with recombinant TGF-β (rTGF-β). **(C)** Representative histogram and **(D)** cumulative data for CD73 expression intensity on splenic CECs untreated or treated with rTGF-β. **(E)** Representative flow cytometry plot and **(F)** cumulative frequency of p-SMAD3+ splenic CECs untreated or treated with rTGF-β. **(G)** Representative flow cytometry plot and **(H)** cumulative frequency of p-SMAD3+ CD39- versus CD39+ splenic CECs. MFI, Mean fluorescence intensity; FMO, Fluorescence minus one. *$P < 0.05$, **$P \leq 0.01$, ***$P \leq 0.001$, and ****$P \leq 0.0001$.

S Fig. 3

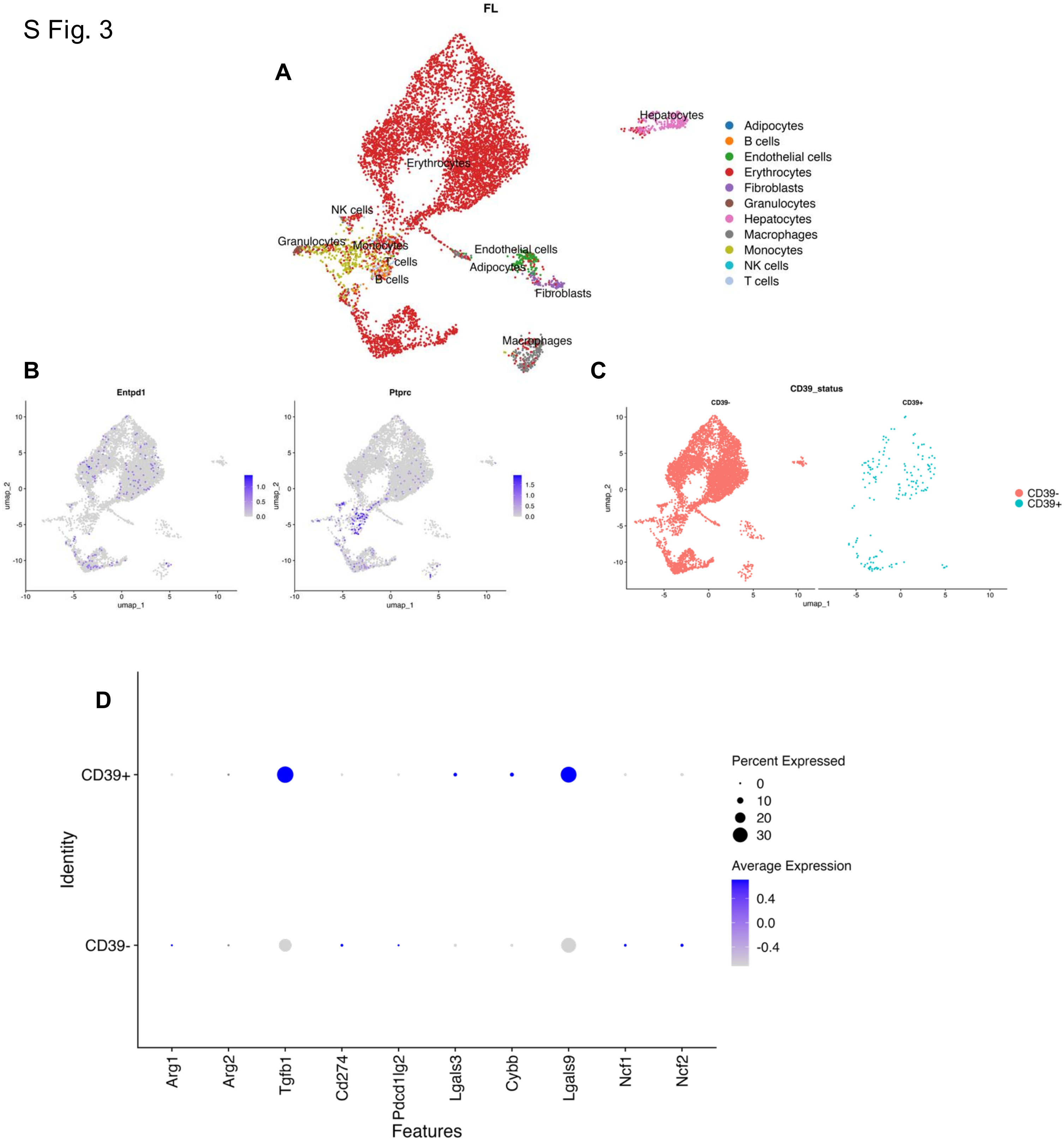

S Fig 3. **(A)** Unsupervised graph-based clustering and Uniform Manifold Approximation and Projection (UMAP) visualization of fetal liver cells. **(B)** FeaturePlot of *Entpd1* and *Ptprc* expression in fetal liver erythroid cells. **(C)** UMAP showing the distribution of CD39- and CD39+ CECs in the fetal liver. **(D)** DotPlot comparing the expression of immunomodulatory gene expression in CD39- and CD39+ CECs from the fetal liver.

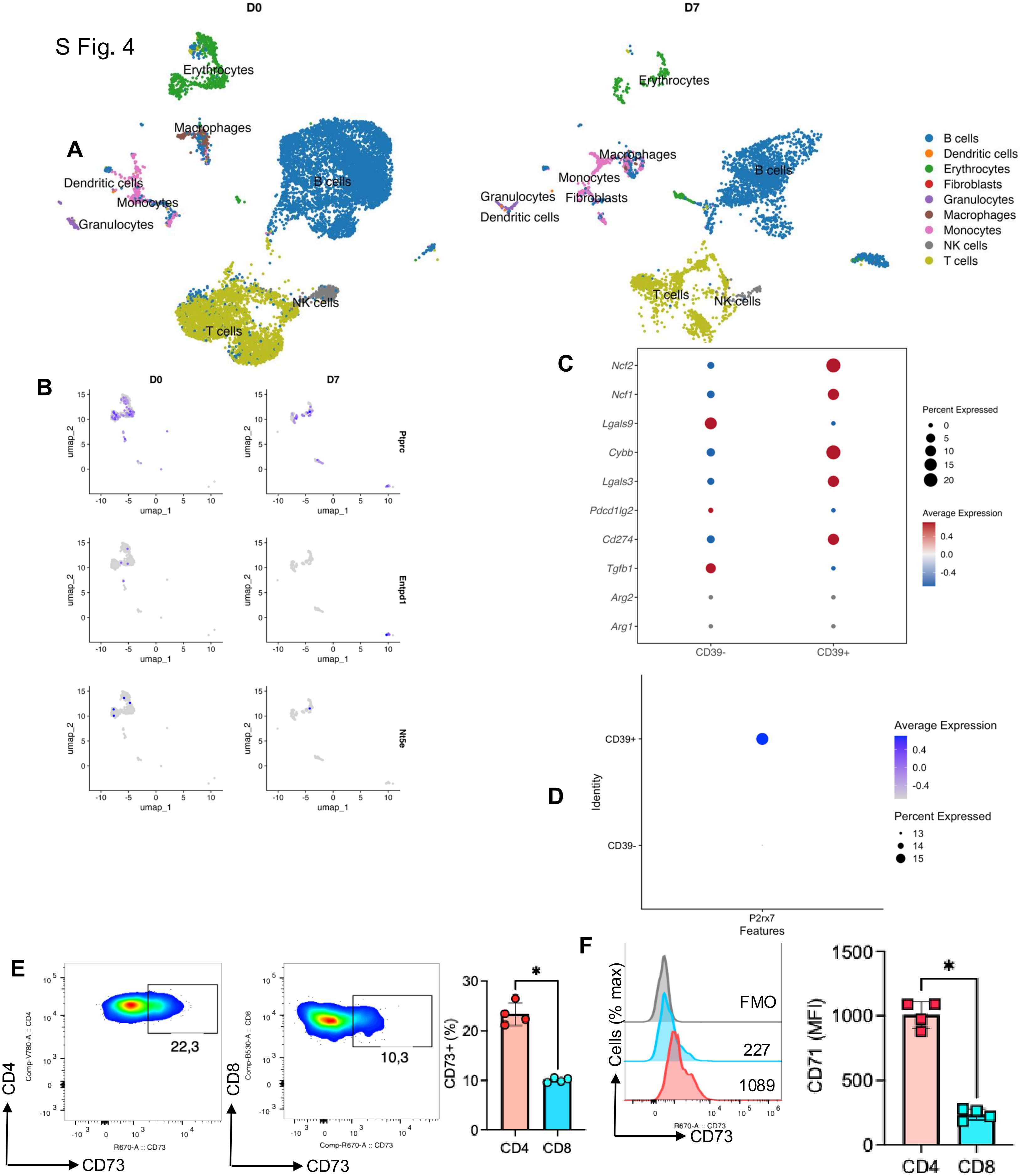

S Fig 4. (A) Unsupervised graph-based clustering and Uniform Manifold Approximation and Projection (UMAP) visualization of splenocytes from C57BL/6J mice at steady state day 0 (D0) and on D7 of *P. chabaudi* infection. **(B)** FeaturePlot of *Ptprc, Entpd1,* and *Nt5e* expression in erythrocytes at steady state (D0) and *P. chabaudi* infection (D7). **(C)** DotPlot comparing the expression of immunomodulatory gene expression in CD39^{-} and CD39^{+} splenic CECs. **(D)** Expression of *P2rx7* in CD39^{-} and CD39^{+} CECs from 4T1-bearing mouse spleen. (**E**) Representative flow plots and cumulative data showing the frequency of splenic CD73^{+} T cells. (**F**) Representative flow cytometry histograms and cumulative data showing the intensity of CD73 expression on T cells measure by mean fluorescence intensity (MFI). Fluorescence minus one (FMO). Eat dot represents data from an animal.
